# In Silico Screening and Identification of Antidiabetic Inhibitors Sourced from Phytochemicals of Philippine Plants against Four Protein Targets of Diabetes (PTP1B, DPP-4, SGLT-2, and FBPase)

**DOI:** 10.3390/molecules28145301

**Published:** 2023-07-09

**Authors:** Mark Andrian B. Macalalad, Arthur A. Gonzales

**Affiliations:** Department of Chemical Engineering, University of the Philippines Diliman, Quezon City 1101, Metro Manila, Philippines; mbmacalalad@up.edu.ph

**Keywords:** diabetes, phytochemicals, natural compounds, PTP1B, DPP-4, SGLT-2, FBPase, ADMET, molecular docking, MD simulations, MM/PBSA

## Abstract

Current oral medications for type 2 diabetes target a single main physiological mechanism. They either activate or inhibit receptors to enhance insulin sensitivity, increase insulin secretion, inhibit glucose absorption, or inhibit glucose production. In advanced stages, combination therapy may be required because of the limited efficacy of single-target drugs; however, medications are becoming more costly, and there is also the risk of developing the combined side effects of each drug. Thus, identifying a multi-target drug may be the best strategy to improve treatment efficacy. This study sees the potential of 2657 Filipino phytochemicals as a source of natural inhibitors against four targets of diabetes: PTP1B, DPP-4, SGLT-2, and FBPase. Different computer-aided drug discovery techniques, including ADMET profiling, DFT optimization, molecular docking, MD simulations, and MM/PBSA energy calculations, were employed to elucidate the stability and determine the binding affinity of the candidate ligands. Through in silico methods, we have identified seven potential natural inhibitors against PTP1B, DPP-4, and FBPase, and ten against SGLT-2. Eight plants containing at least one natural inhibitor of each protein target were also identified. It is recommended to further investigate the plants’ potential to be transformed into a safe and scientifically validated multi-target drug for diabetes therapies.

## 1. Introduction

Type 2 diabetes (T2D) is a chronic condition in which the body becomes insulin resistant or does not produce enough insulin, leading to elevated blood glucose levels [1]. There is no known cure for T2D, but it can be managed through lifestyle changes, such as diet, exercise, and oral medications [2,3]. If left uncontrolled, T2D may lead to several health complications, including kidney failure, heart attacks, stroke, lower limb amputation, and adult-onset blindness [4]. It is estimated that over 500 million people worldwide have type 2 diabetes, and this number is expected to rise to 783 million by 2045 [5]. Despite efforts to increase awareness of diabetes prevention and risk factors, morbidity and mortality rates continue to rise globally. The increasing prevalence of diabetes highlights the insufficiency of currently available medications and implores the need for more alternative measures to improve current treatment options.

Oral hypoglycemic agents used to manage T2D employ various mechanisms of action that trigger different physiological responses; they either activate or inhibit drug receptors to improve insulin sensitivity, increase insulin secretion, block glucose absorption, or decrease glucose production [6]. The current drugs, however, have been linked to the development of undesirable side effects such as hypoglycemia, weight gain, nausea, gastrointestinal disturbances, hepatoxicity, and diarrhea [3,6]. In advanced stages of T2D, combination therapy with two or more oral drugs from different classes that target multiple processes may be necessary for optimal management because of the limited efficacy of monotherapy or single-target drugs [7]. Moreover, as the cost of synthetic medications continues to rise, patients have limited access to affordable treatment options, making diabetes more challenging to treat, especially in developing countries [8]. Since diabetes requires long-term treatment, there is always a high demand for a safe and cost-effective antidiabetic drug; hence, identifying and developing a multi-target drug may be the most effective strategy to enhance diabetes therapies.

This study selected four proteins as potential therapeutic targets, with each protein associated with one specific physiological response. Protein tyrosine phosphatase 1B (PTP1B) is a highly specific enzyme in the endoplasmic reticulum (ER) that catalyzes the dephosphorylation of activated insulin receptors (IR) and insulin receptor substrates (IRS), attenuating insulin signaling transduction. The dephosphorylation of IR and IRS impedes the downstream processes, such as the translocation of the glucose transport protein (GLUT4) to the cell membrane, which is required for glucose molecules to move out of the bloodstream [9,10]. Despite recognizing PTP1B’s importance as a therapeutic target, there are still no approved drugs against PTP1B [11]. Dipeptidyl peptidase 4 (DPP-4) is an enzyme that inactivates incretin hormones, mainly glucagon-like peptide-1 (GLP-1) and gastric inhibitory peptide (GIP), whose primary function is to increase insulin secretion. Therefore, inhibiting the DPP-4 enzyme would protect the incretins from being destroyed, leading to more incretins to stimulate insulin secretion [12,13]. Sodium–glucose co-transporter 2 (SGLT-2) is a transport protein that facilitates 90% of glucose reabsorption in the kidney back to the bloodstream. Inhibition of SGLT-2 activity is an ideal approach to managing diabetes since it prevents a large amount of glucose from being reabsorbed into the human body. Once the transfer of sugar molecules is inhibited, the excess glucose in the kidney is eliminated through the urine [14,15]. The last protein to be targeted is fructose 1,6-bisphosphatase (FBPase). It is a key rate-limiting enzyme of gluconeogenesis that converts non-carbohydrate substrates, such as lactates and pyruvates, into glucose. FBPase catalyzes the hydrolysis of fructose-1,6-bisphosphate to fructose-6-phosphate, a precursor compound for the formation of free glucose [6,16]. FBPase has been recently viewed as a viable molecular target for regulating sugar levels, and inhibitors against this enzyme may address the unmet need for diabetes therapies [17].

The rising cost and side effects of synthetic medications have rekindled the interest in traditional medicine, particularly herbal plants, as a potential treatment option [8]. Medicinal plants are valued in traditional medicine for their safety, accessibility, and efficacy [18,19]. They are a rich source of phytochemicals—the diverse chemical compounds produced by plants—that have the potential to be utilized directly or as structural templates for creating new and more potent medications [20]. With regard to diabetes, numerous phytochemicals have been reported to show antidiabetic activity with varying mechanisms of action, which may be suitable for the multi-target drug approach [21]. Ethnobotanical information from studies reported over 1200 plant species possessing antidiabetic potential [22], but most lack a mechanism-based study to provide full scientific support for these natural compounds [23]. Despite the immense potential of natural products, their use in drug discovery has diminished over the past two decades due to the technical barriers in conventional screening assays. With the advancements in computational chemistry, including new tools in computer-aided drug design (CADD) and faster computers, the interest in phytochemicals as drug leads has been revitalized [24]. CADD approaches such as molecular modeling and in silico simulations allow the analysis and visualization of the interaction between lead compounds and their targets in a short time, even before drug synthesis and in vitro or in vivo experiments. This accelerates the drug discovery process by reducing the time, labor, and cost associated with traditional experimental methods [25].

## 2. Results

### 2.1. ADMET Profiling

Of the 2657 ligands tested, only 373 passed the ADMET filter. The numbers of candidate ligands that passed each parameter are presented in Table 1, and the list of the compounds that scored favorably in the ADMET profiling is provided in the Supplementary Materials (Table S1).

Human Oral Bioavailability was the strictest parameter, eliminating almost 63% of natural compounds. About 25% cannot be absorbed into the bloodstream through the gastrointestinal system. Over 50% may cause hepatic toxicity, while nearly 26% may be carcinogenic. Only nine ligands violated the Acute Toxicity Rule, which is expected, since most phytochemicals are generally safe to be consumed [26]. As for the references used in this study, the majority showed signs of hepatoxicity and carcinogenicity, even for drugs including ALO, SIT, VIL, CAN, and EMP, which are already approved on the market (Table S2). MB0, a reference drug for FBPase, did not meet the criteria of Lipinski’s Rule of Five and also showed poor results in the Human Intestinal Absorption and Human Oral Bioavailability tests. These factors could be why MB0 is still being investigated in clinical trials [27]. With these observations, candidate ligands that passed our ADMET filter may have lower health risks and better bioavailability than currently available options.

### 2.2. DFT Optimization of Ligands

Prior to molecular docking experiments, the structures of the 373 ligands and 12 references were optimized using DFT by obtaining the structure that gave the lowest possible ground state energy. The structures from PubChem had minor structural changes since compounds stored in the PubChem library were already standardized with the IUPAC International Chemical Identifier (InChI) software version 1.06 [28], and the 3D models were already optimized using the Merck Molecular Force Field (MMFF94s) [29].

### 2.3. Molecular Docking

Consensus docking was implemented to improve docking performance, because it increases the ranking power and hit rates by combining the information about the predicted binding modes from two different scoring functions to generate a consensus prediction of the most likely binding pose of the ligand. This method eliminates the limitations and biases of individual docking algorithms and provides a more accurate prediction of the ligand binding mode [30]. Since the scoring functions of Autodock 4.2.6 [31] and Autodock Vina 1.2.3 [32] are different, there is an apparent disparity in the scores obtained from each software, and the rankings produced by the two docking programs are not always consistent with each other. Nonetheless, the ligands in the top 10 positions in both software programs were among the highest-scoring candidates. The complete docking scores of all ligands are presented in Table S3 of the Supplementary Materials, while the structures of the top 10 ligands with the highest docking scores are presented in Figures S1–S4.

There were no common top ligands in all four proteins (Table 2). However, it was observed that C-1254 and C-1717 exhibited promising characteristics for three protein targets. C-1254 displayed favorable interactions against PTP1B, SGLT-2, and FBPase, suggesting a potential for multi-target inhibition. Similarly, C-1717 showed potential for targeting PTP1B, DPP-4, and SGLT-2. Furthermore, several other phytochemicals, namely, C-0310, C-1190, C-1287, C-1939, C-2082, and C-2083, were among the top compounds of two proteins. The interactions of the top ligands against each protein are discussed in the next sections.

#### 2.3.1. PTP1B

**Figure 1:**
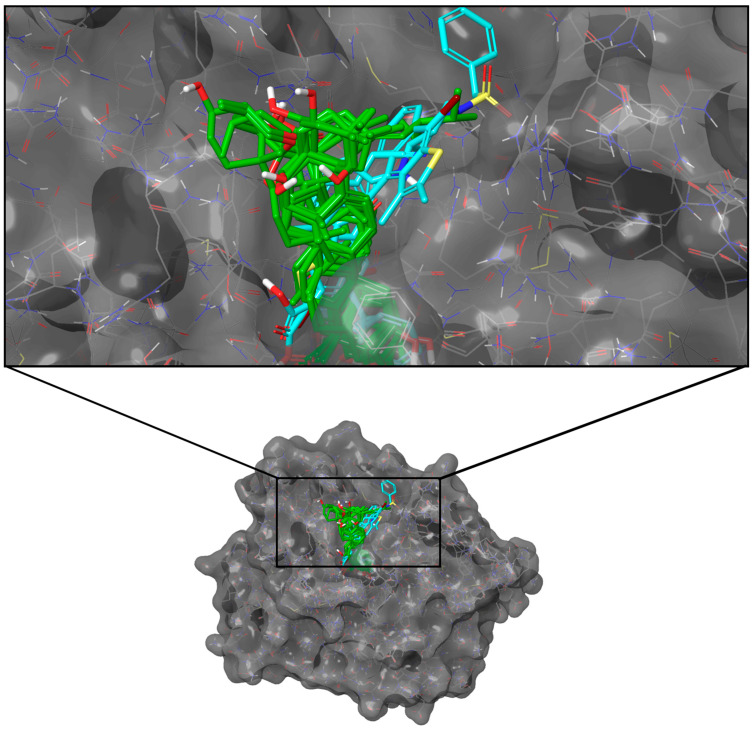
Docking poses of the top ten ligands and the three controls against the binding region of PTP1B (PDB ID: 2QBP). Candidate ligands are shown in green, while references are in cyan.

Only seven ligands surpassed the consensus docking score of KQ7 (−75.77 kJ/mol), with C-1823 obtaining the highest overall consolidated score (Table 2). None recorded a higher docking score than the reference R86 (−96.61 kJ/mol). All of the top ten ligands had four to six rings with polar hydroxyl and carbonyl groups and formed similar poses within the binding pocket (Figure 1). 

**Figure 2:**
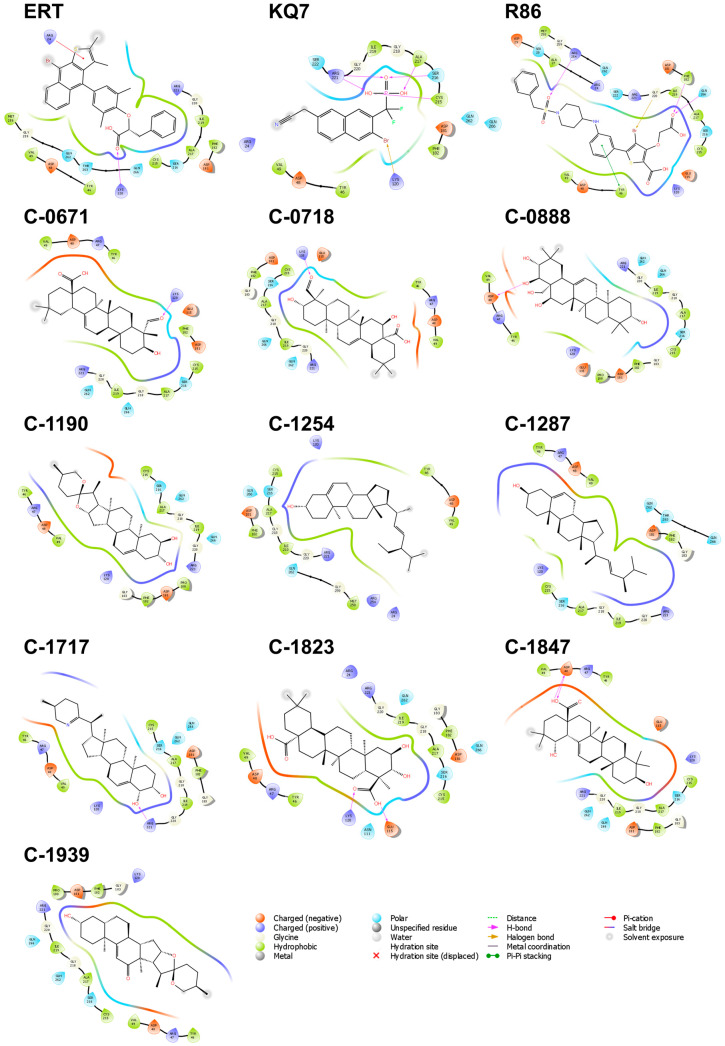
Residue interactions within 4.0 Å against the binding pocket of PTP1B. The majority of the interactions are hydrophobic and electrostatic. Most H-bonds are formed with Lys 120, Asp 48, and Arg 221.

The interactions within the binding pocket of PTP1B consist mainly of hydrophobic and charged, suggesting that the binding region includes a combination of both polar and non-polar residues (Figure 2). Eleven residues, Ala 217, Arg 221, Asp 181, Cys 215, Gln 266, Gly 220, Ile 219, Lys 120, Ser 216, Tyr 46, and Val 49, were found to have interacted with all the ten candidate ligands and the three references. Among them, Cys 215, Asp 181, and Arg 221 hold important roles in the catalytic activity of the protein [30]; thus, the interactions observed on these three residues could potentially improve the binding and effectiveness of the proposed ligands. The catalytic domain of PTP1B can be divided into four sites, one catalytic site (A-site), the most accessible binding site in the protein, and three neighboring non-catalytic sites (B-site, C-site, and D-site), which are critical for the selectivity of PTP1B inhibitors [30]. C-1823, C-1254, and the three references established contact with the B-site residues of the catalytic domain, including Arg 24, Arg 254, Met 258, and Gly 259. The added interactions in the secondary binding site increase the potency and specificity of the inhibitors. Specifically, hydrogen bond interactions with Arg 24 and Arg 254 of the B-site may result in a 20-fold increase in selectivity [30,31]. It is also interesting that Lys 120, Tyr 46, and Val 49 are among the highly interacted residues, even when these three are not part of the primary catalytic domain. Lys 120 is a critical residue in the D-site, Tyr 46 is part of the C-site residues, and Val 49 borders the residues of the B-site. Aside from the heightened potency, interaction with Lys 120 also improves the compound’s selectivity over other protein tyrosine phosphatases (PTP) [33]. Since the other members of the PTP enzymes only differ from PTP1B by a few amino acids in the B-, C-, and D-sites, forming strong interactions within these secondary subsites would be critical in improving the selectivity of potential PTP1B inhibitors.

#### 2.3.2. DPP-4

**Figure 3:**
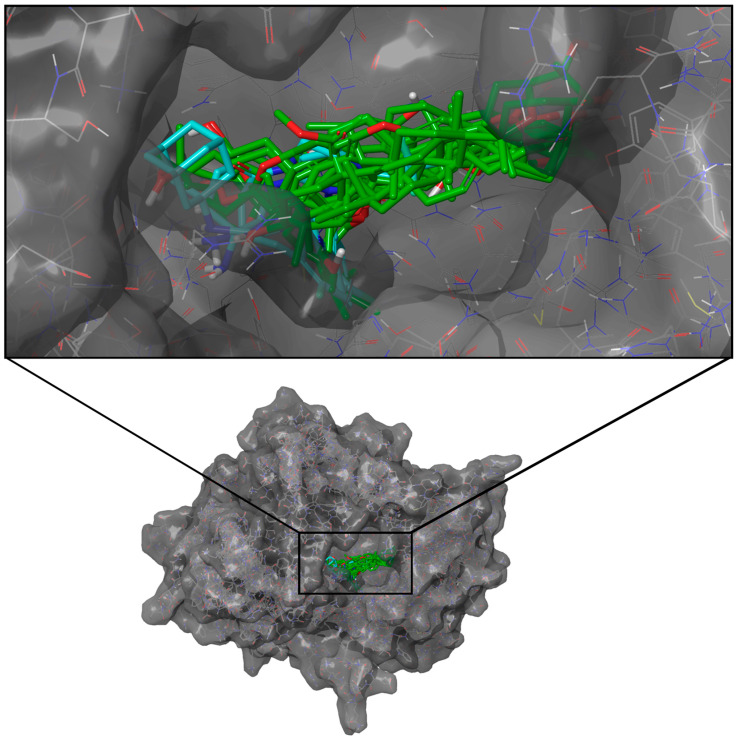
Docking poses of the top ten ligands and the three controls against the binding region of DPP-4 (PDB ID: 2ONC). Candidate ligands are shown in green, while references are in cyan.

All top ten candidate ligands against DPP-4 scored higher than the two references, SIT (−75.44 kJ/mol) and VIL (−70.33 kJ/mol), but only three ligands, namely, C-1939, C-1968, and C-2186, scored higher than ALO (−80.79 kJ/mol) (Table 2). The binding pocket of DPP-4 is situated inside a deep cave where the 10 candidate ligands and the references adapted into similar binding poses (Figure 3).

**Figure 4:**
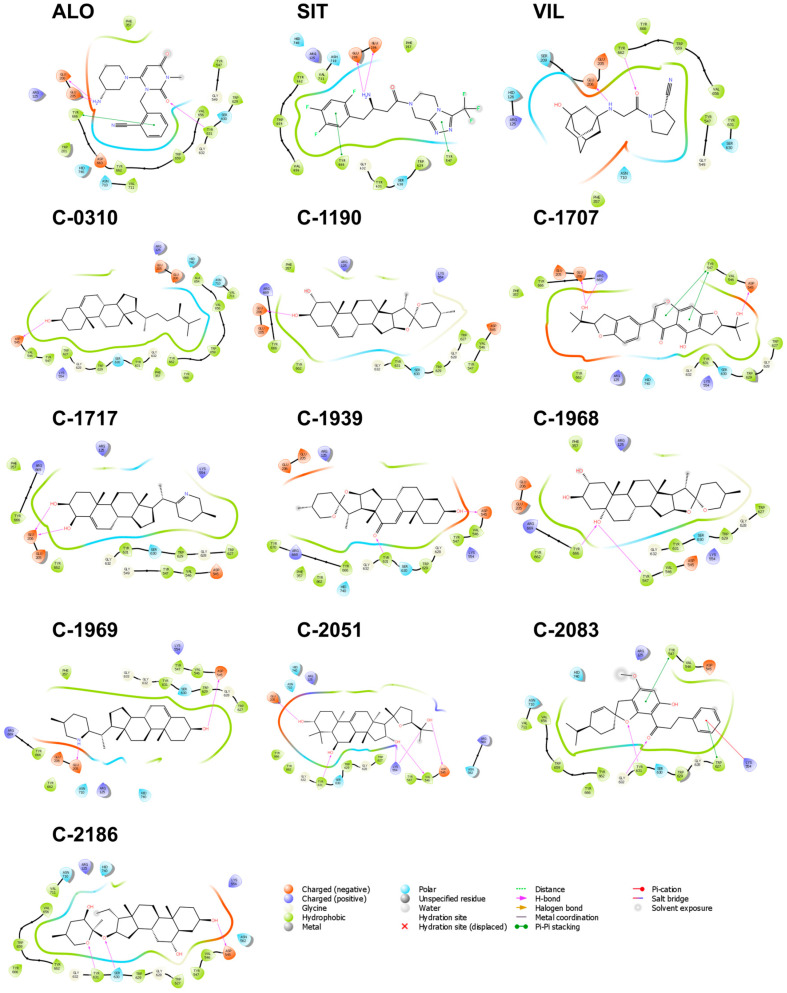
Residue interactions within 4.0 Å against the binding pocket of DPP-4. The majority of the interactions are hydrophobic, while a few charged and polar interactions are also present. Most H-bonds are formed with Glu 206, Glu 205, and Asp 545.

The majority of the interacting residues of DPP-4 were hydrophobic, with a few polar and charged residues (Figure 4). Six residues, Arg 125, Tyr 547, Ser 630, Tyr 631, Tyr 662, and Tyr 666, consistently interacted with all the reference and the candidate ligands—four of which are hydrophobic: Tyr 547, Ser 630, Tyr 631, and Tyr 666. Studies have pointed out that the cumulative binding energies from the hydrophobic interactions drive the efficacy and enhanced activity of DPP-4 inhibitors [34,35]. Of particular interest is Ser 630. It is one of the catalytic triads essential for catalytic activity; hence, forming strong interactions with this residue will improve the inhibitor’s potency. All candidate ligands interacted with Ser 630; however, only the ligand C-2186 established H-bond connections with the residue. Polar interactions with His 740, also one of the catalytic triads, were observed with C-0310, C-1707, C-1939, C-1969, C-2051, C-2083, and C-2186, but interactions with Asp 708, another member of the catalytic triad, were more elusive and formed zero contacts with any of the ligands. It has also been found that mutations to Tyr 547, Glu 205, and Glu 206 decrease enzymatic activity [36,37]; therefore, aside from the catalytic triad, Tyr 547, Glu 205, and Glu 206 are equally viable targets. Tyr 547 formed pi–pi stackings with C-1707, C-2083, and SIT. It also made H-bond contacts with C-1969. Interactions with Glu 205 and Glu 206 are also crucial for the activity of the DPP-4 inhibitors (ALO and SIT). Both drugs exhibit high potency in inhibiting DPP-4 activity, as they form strong interactions with Glu 205 and Glu 206 [38]. Hence, the interaction of other candidate ligands with Glu 205 and Glu 206 may also suggest a heightened inhibitory activity.

#### 2.3.3. SGLT-2

**Figure 5:**
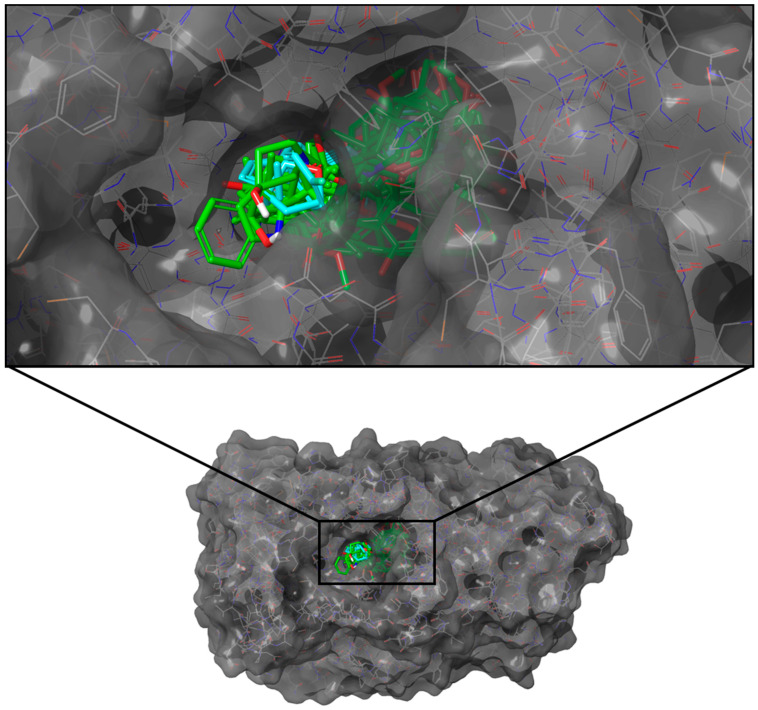
Docking poses of the top ten ligands and the three controls against the binding region of SGLT-2 (PDB ID: 7VSI). Candidate ligands are shown in green, while references are in cyan.

None of the top-performing compounds obtained a more negative score than the consolidated docking score of CAN (−96.06 kJ/mol). Only two compounds, C-1254 and C-2084, surpassed EMP’s docking score (−92.17 kJ/mol), but all ten compounds scored higher than DAP (−87.11 kJ//mol) (Table 2). The ten ligands and the three references have similar elongated structures, with three to five rings and a few hydroxyl groups. The ligands are docked within a small protein cavity and feature analogous binding conformations (Figure 5).

**Figure 6:**
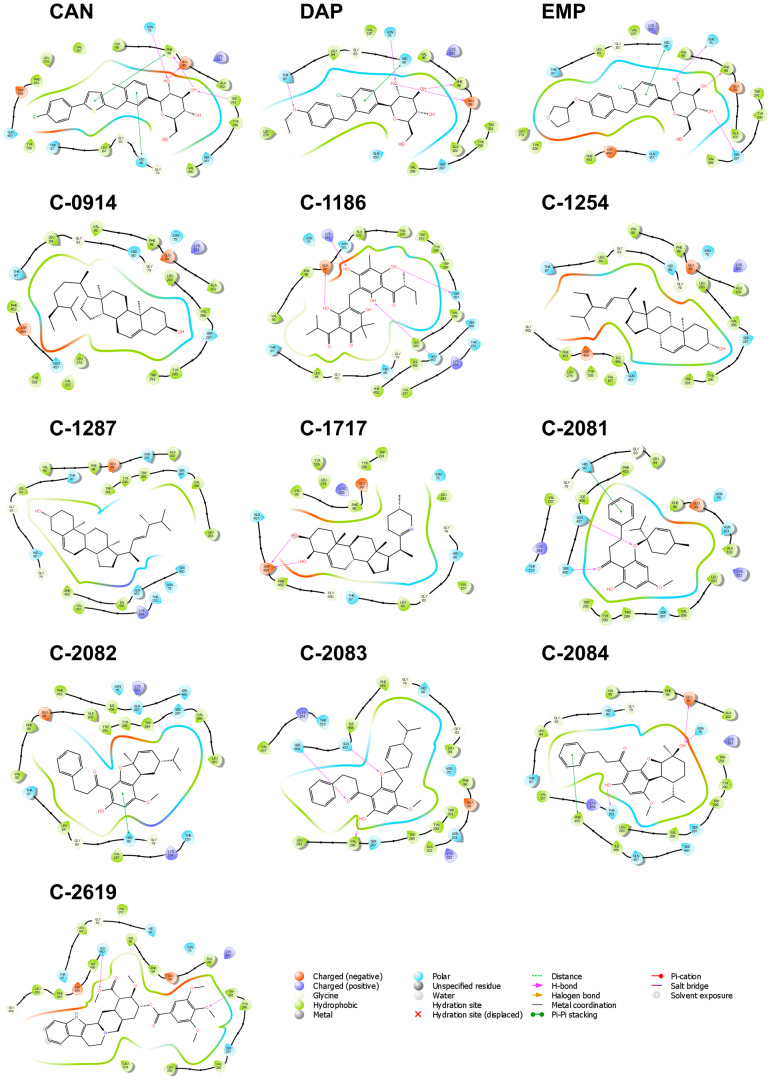
Residue interactions within 4.0 Å against the binding pocket of SGLT-2. The most common type of interaction observed was hydrophobic interactions, with a few polar interactions. H-bonds mostly occurred with Asn 75, Glu 99, and Gln 457.

The most common interaction type observed is hydrophobic, followed by a few polar interactions (Figure 6). Charged or electrostatic interactions were also observed for Lys 321 and Lys 154. Ten residues, Gln 457, Glu 99, Gly 83, His 80, Leu 84, Phe 98, Trp 291, Tyr 290, Val 157, and Val 286, formed interactions with all ten of the highest-scoring ligands and the three references. Among these, Phe 98 and Val 157 play essential roles in the inhibition activity of the drug Empagliflozin, and any mutation to these residues can decrease the inhibitor’s potency and selectivity [39], so the interaction of Phe 98 and Val 157 with all the candidate ligands highlights their importance in the binding mechanism. Other vital residues to the binding activity include Phe 453, Val 95, and Leu 283 [39].

Additionally, polar interactions occurred between the hydroxyl groups of the ligands and two of the eleven residues: Gln 457 and His 80. These polar interactions are also considered important for SGLT-2 inhibition [39]. Aside from the polar contacts, His 80 also formed pi–pi stacking with CAN, DAP, EMP, C-2081, and C-2082, while Gln 457 H-bonded with C-2081, C-2083, and C-2619. Other residues that interacted with all candidate ligands during binding were Gly 83, Leu 84, Glu 99, Val 157, Trp 290, and Tyr 291. These residues do not directly affect Empagliflozin’s potency [39] but may present significant roles in the stability and affinity of the candidate natural compounds. 

#### 2.3.4. FBPase

**Figure 7:**
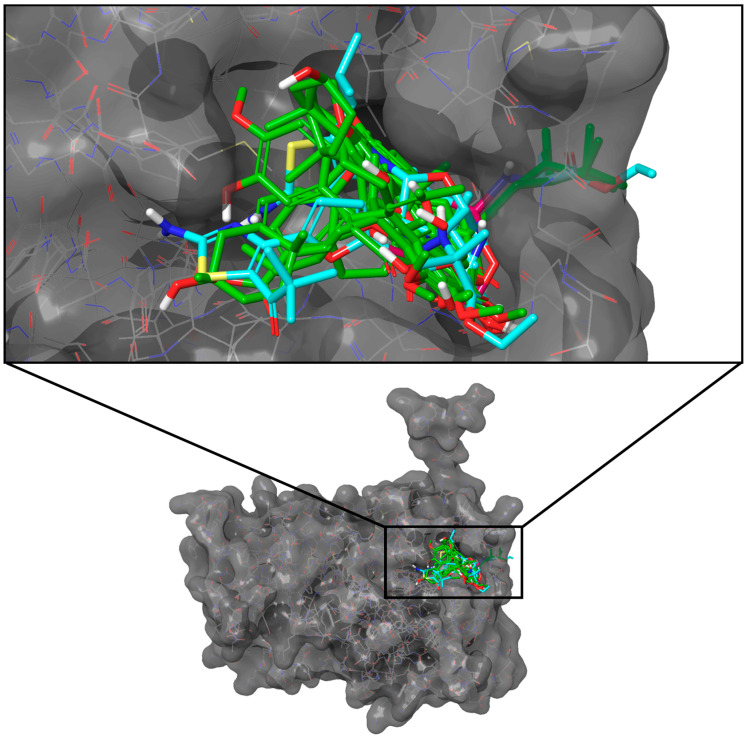
Docking poses of the top ten ligands and the three controls against the binding region of FBPase (PDB ID: 2FIE). Candidate ligands are shown in green, while references are in cyan.

All ten compounds outperformed the three control compounds’ consolidated docking scores (−58.87 kJ/mol to −60.46 kJ/mol), with C-0829 achieving the highest score (−67.53 kJ/mol) (Table 2). The top-scoring ligands against FBPase had the most structurally diverse compounds, further reflected in their unique and distinct docking poses (Figure 7). 

**Figure 8:**
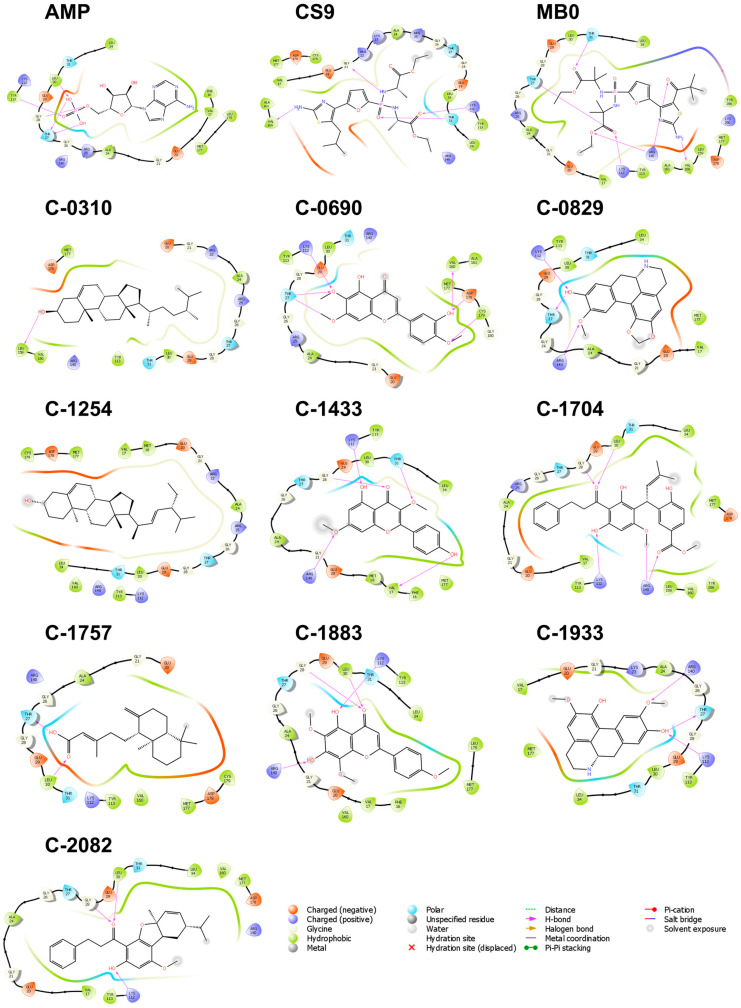
Residue interactions within 4.0 Å against the binding pocket of FBPase. Hydrophobic, charged, and polar interactions are the most frequent interaction types. Thr 27, Thr 31, Lys 112, and Arg 140 formed multiple H-bond interactions.

Hydrophobic interactions were the most common type of interaction; however, charged and polar interactions were also frequent (Figure 8). The amino acids arginine, lysine, glutamic acid, and aspartic acid all contributed to bridging the ligands to the binding site through electrostatic contact. There are also a few polar interactions from Thr 31 and Thr 27. 

Given that the AMP allosteric binding site is predominantly hydrophilic [17], it was expected that polar and charged amino acids would be more involved in the binding, as seen in Figure 8. A study also revealed that polar interactions were more important than hydrophobic interactions, validating our observations [40]. Twelve residues formed connections to all candidate ligands and the three references: Ala 24, Arg 140, Glu 29, Gly 21, Gly 26, Leu 30, Met 177, The 27, Thr 31, and Tyr 113. Of these 12, Met 177, Thr 31, and Tyr 113 are considered the most important residues due to their negative effect on the binding affinity when they are mutated [41]. Lys 112 is also among the key residues [41] and only missed interacting with C-0310.

Several hydrogen bond pairs were formed between the critical residues and the candidate ligands. Lys 112 paired with C-0690, C-0829, C-1433, C-1704, C-1883, C-1933, C-2082, CS9, and MB0; Tyr 113 with AMP; and Thr 31 with C-1433, CS9, and MB0. The compound C-1254 was the only one that did not form any H-bond pairs due to the compound’s lone hydroxyl group. Despite the absence of any hydrogen bonds, C-1254 still had a favorable docking score.

### 2.4. Molecular Dynamics Simulations

**Figure 9:**
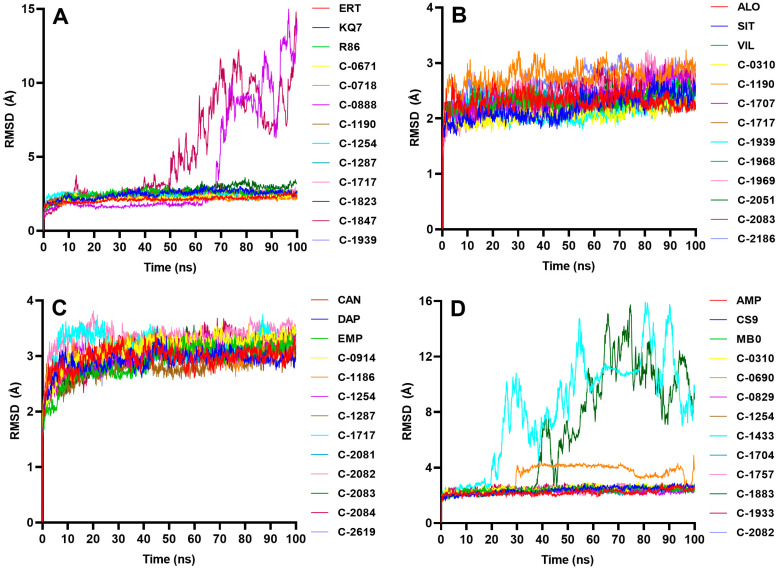
Complex RMSD of the highest-scoring candidate ligands against the four protein targets: (**A**) PTP1B, (**B**) DPP-4, (**C**) SGLT-2, (**D**) FBPase. C-0888 and C-1847 presented instability against PTP1B, while C-0690, C-1433, and C-1883 exhibited unstable behaviors with FBPase. The rest stabilized at around 30–40 ns after system equilibration.

**Figure 10:**
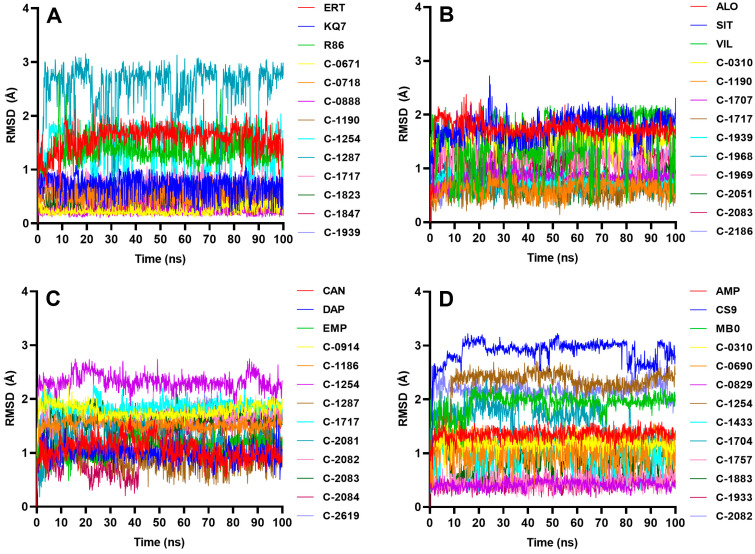
Ligand–RMSD of the highest-scoring candidates’ ligands against the four targets: (**A**) PTP1B, (**B**) DPP-4, (**C**) SGLT-2, (**D**) FBPase. For PTP1B systems, only C-1287 had discernable pose changes throughout the 100 ns run; however, the ligand was still fully bound inside the binding pocket, even with multiple conformational movements. The control compound VIL against DPP-4 also took on a variety of poses but remained intact inside the binding cavity. A slight rise in the ligand–RMSD at the equilibration phase was observed with C-0914, C-1254, C-1717, C-2082, C-2083, and C-2084 of the SGLT-2 complexes. As for the FBPase complexes, the reference CS9 had the most movement, due to its highly rotatable structure.

**Figure 11:**
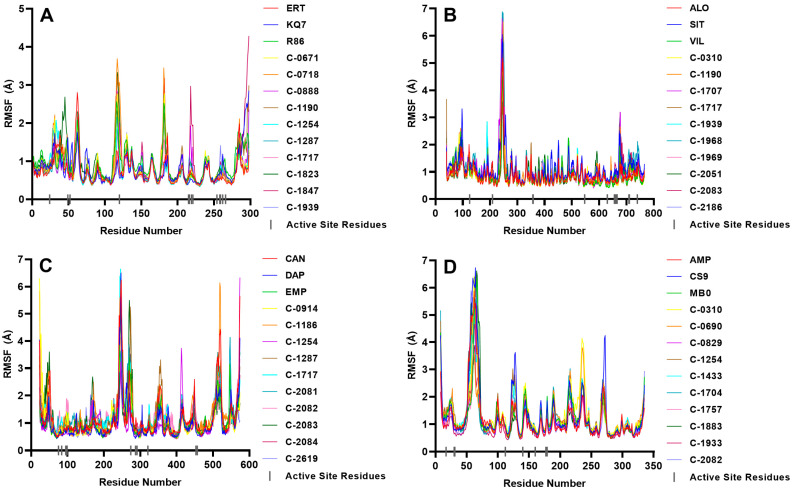
RMSF of the highest scoring ligands against the four protein targets: (**A**) PTP1B, (**B**) DPP-4, (**C**) SGLT-2, (**D**) FBPase. The grey bars at the x-axis mark the location of the known important residues.

The stability of the protein–ligand complex was analyzed during MD simulations, and the complex’s conformational response over time due is reflected in the RMSD and RMSF trajectories. Water, chlorine, and sodium ions were included in the all-atom MD simulations, but only the complex–RMSD (Figure 9), ligand–RMSD (Figure 10), and protein–RMSF (Figure 11) were examined to observe the stability of the docked ligands.

**Figure 12:**
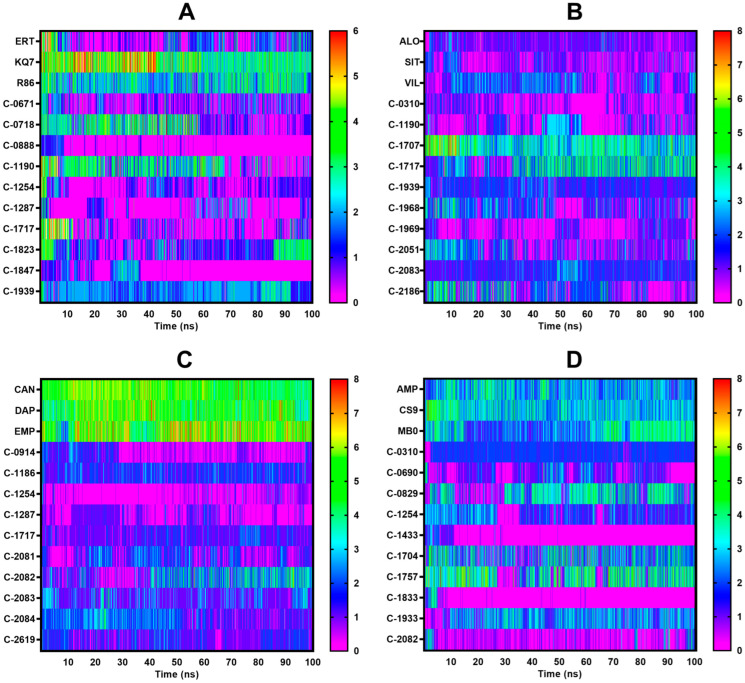
H-bond diagram for all four protein complexes. (**A**) PTP1B, (**B**) DPP-4, (**C**) SGLT-2, (**D**) FBPase. C-0888 and C-1847 paired with PTP1B, C-0690, and C-1433, and C-1833 paired with FBPase failed to bind to the protein, resulting in virtually zero H-bonds toward the end of the simulation.

C-1254 and C-1717, two of the top ten candidate ligands for multiple proteins, demonstrated stability with their respective targets (Figure 9). C-1254 exhibited a stable behavior when interacting with PTP1B, SGLT-2, and FBPase, while C-1717 also maintained its stability with PTP1B, DPP-4, and FBPase. C-0310, C-1190, C-1287, C-1939, C-2082, and C-2083, which targeted up to two proteins, also presented stable behaviors. These ligands displayed minimal conformational changes throughout the entire simulation run, as depicted in the ligand–RMSD plots in Figure 10. Furthermore, the consistent number of hydrogen bonds (Figure 12) with their respective targets contributed to the observed stability.

#### 2.4.1. PTP1B

Eight ligands demonstrated structural stability similar to the reference compounds (Figure 9), while the remaining two failed to bind stably to the protein. A short clip of a stable PTP1B complex can be observed in Video S1 in the Supplementary Materials. C-0888 initially exhibited a stable behavior during the simulation, but, at around 60 ns, the ligand dissociated from the binding site. Similarly, C-1847 showed signs of stability, but the RMSD rose sharply after a few nanoseconds, indicating that the ligand had separated from the active site. Visual inspections of Videos S2 and S3 also show how C-0888 and C-1847 separated from the binding site. Looking at the ligand–protein interaction diagram from Figure 2, C-0888 has similar interactions with C-0718, and both have identical binding poses. However, the latter formed a stable conformation, while C-0888 detached from the binding site and moved around the protein. One of the differences is the hydrogen bond C-0718 formed with the residue Lys 120 (Figure 2). Lys 120 is a known amino acid residue of PTP1B that significantly improves the potency and selectivity of the ligand [33], so the added Coulombic interaction may have offered a significant increase in its binding strength. C-0888 and C-1847 also contain hydroxyl groups exposed to the solvent, making them more susceptible to forming hydrogen bonds with water molecules. This reduces the binding strength between the ligands and the protein and increases the likelihood of the solvent pulling the ligand out of the binding pocket.

Figure 10 displays the ligand pose changes during the 100 ns simulation. A constant and low ligand–RMSD indicates that the ligand structure during the simulation remains similar to its docking pose, while a fluctuating RMSD suggests that the ligand frequently alters its pose in the binding pocket. Most candidate ligands maintained their docking poses, except for C-1287, which constantly changed its position but still bound stably to the protein. Meanwhile, C-0888 and C-1847, the two ligands that failed to stabilize with the protein, retained their docking positions despite being exposed to water molecules, since their compact structures restricted the ligands from undergoing significant structural changes. 

RMSF plots show which part of the protein constantly moves throughout the 100 ns simulation. The eight stable PTP1B complexes showed low RMSF values in their most important residues (Figure 11), indicating minimal movements in the binding region and stable ligand adherence. However, the complexes with C-0888 and C-1847 had high RMSF values on key residues Cys 215, Ser 216, Ile 219, and Arg 221. Cys 215 and Arg 221 are crucial for binding in the primary pocket [42], and the ligand’s inability to bind to these residues negatively affected the complex’s stability. This observation emphasizes the importance of these residues in the protein’s catalytic activity.

Another tool to assess stability is the H-bond diagram shown in Figure 12. At the beginning of the equilibration phase, all ligands, including the references, had more H-bonds, but the number decreased as the ligand adapted to its environment. C-0718, C-1190, C-1823, and C-1939 formed the most H-bonds, while C-1287 formed the fewest due to constantly changing conformation and its single hydroxyl group, limiting its ability to form H-bonds. Ligands that failed to stabilize with PTP1B experienced a drastic decrease in H-bonds towards the end of the simulation since they were absorbed by the solvent, preventing them from forming H-bonds with the active site. 

#### 2.4.2. DPP-4

All 10 candidate ligands presented a stable behavior when simulated with the DPP-4 protein (Figure 9). The 10 complexes quickly adjusted to their new environment within 20 ns and stabilized without significant fluctuations, as seen in Video S4 of the Supplementary Materials. The ligands were strategically docked within the binding site of DPP-4, where the polar groups (i.e., hydroxyl groups) were conveniently surrounded by polar amino acids and some charged amino acids, offering additional attractive forces (Figure 4). The top 10 ligands, except for C-0310, are also rich in functional groups (Figure S2), which are capable of H-bonding to solidify the binding. Aside from H-bond pairs, the pi–pi stacking observed on the ligands C-1707 and C-2083 may have also helped stabilize the ligands.

The erratic fluctuations observed in the reference VIL (Figure 10) are caused by the rapid movements of the hydroxyl-containing adamantyl group, which pivots at the middle nitrogen of the ligand (Figure S2). The hydroxyl group of the adamantyl is situated near positively charged amino acids that cannot form dipole interaction nor H-bonds with the free OH^−^ group of the ligand. Nevertheless, the ligand stayed inside the binding pocket due to hydrophobic interactions and H-bonds formed with the middle nitrogen and carbonyl group.

Active site residues showed low RMSF values (Figure 11), indicating that the receptor site was not altered during movement with the ligand. A peak was observed in residues 240–260 due to a beta-sheet loop exposed to the solvent (Figure S5), but it was far from the active site and did not affect the ligand’s overall binding. This particular protein region may have inadequate connections with the bulk of the protein, causing it to move with the mobile water molecules. 

All ligands had a stable number of H-bonds during the simulation. Still, some experienced a gradual drop in H-bonds as the simulation progressed, potentially due to competing H-bond pairs with water molecules (Figure 12). C-1707, C-1717, C-1939, and C-2083 had the highest number of steady H-bond interactions, while C-0310 had the fewest, due to only having one hydroxyl group for H-bonding.

#### 2.4.3. SGLT-2

The binding region of SGLT-2 is mainly composed of non-polar residues, which primarily contributed to the stable behavior of all the candidate ligands against SGLT-2 through van der Waals and hydrophobic interactions (Figure 9). The ligand is prevented from interacting with water molecules by hydrophobic interactions due to the proximity of the ligand’s lipophilic moieties and the non-polar side chains of the amino acids. At the same time, the induced van der Waals interactions from neutral atoms in the binding pocket kept the ligand in place. Van der Waals forces are considered weak but produce a powerful ligand-binding effect from the collective forces of the surrounding non-polar residues [43]. The synergistic action between the van der Waals forces and hydrophobic interactions overall promotes the binding observed between the ligands and the SGLT-2 protein.

Most top candidate ligands, including references, quickly adopted their desired poses at the start of the simulation and maintained them for the rest of the 100 ns run (Figure 10). Video S5 in the Supplementary Materials shows how the candidate ligand rapidly acclimated to its stable position shortly after equilibration. Some ligands, including C-0914, C-1254, C-1717, and C-2083, had a slight conformational change at the beginning, reflected by small ligand–RMSD rise. C-2082 and C-2084 started with a different conformation but shifted to their definitive binding pose after around 40 ns.

The critical residues remained restricted inside the binding pocket, and no significant deviations occurred to change the active site’s shape (Figure 11). The peaks at opposite ends of the RMSF plot are from the protein’s loose N-terminus and C-terminus, which do not affect ligand–protein interaction. There are also noticeable high peaks from residues 243–253, 268–278, 353–363, and 515–225, but they are far from the binding site. These peaks are from the four nondescript loops of the protein that connect two alpha helices and interact with the mobile water solvent (Figure S6) but played no role in the binding process.

None of the 10 potential ligands formed more H-bonds than the reference compounds (Figure 12). C-1186, C-2082, and C-2083 formed the most H-bond pairs, with two to three pairs each. C-2084 had the most H-bond connections initially, but the number decreased during the simulation. C-0914, C-1254, and C-1287 only had one functional group capable of H-bonding; hence, a low number of H-bonds pairs were found in these ligands. However, these interactions were consistent and contributed to the ligands’ stability in the binding pocket.

#### 2.4.4. FBPase

Seven of the ten ligands against FBPase achieved a stable binding pose with the protein after around 20 ns (Figure 9, Video S6), while the RMSD trajectories of C-0690, C-1433, and C-1883 failed to reach a constant RMSD value, indicating an unstable interaction with the protein (Figure 9). Short clips of the unstable complexes are presented in Videos S7–S9 of the Supplementary Materials. Although the RMSD of C-0690 appeared to approach a constant value, it detached from the binding site after about 30 ns and reattached to a region different from the AMP binding site. At around 90 ns, the ligand completely separated from the protein (Video S7). The three unstable ligands are flavanones with two phenyl groups at opposing ends, containing hydroxyl and methoxy groups and a cyclic ketone with an oxygen atom embedded in the middle (Figure S4). Upon docking, the phenyl group containing most of the oxygen-rich moieties was observed to be directed away from the binding site (Figure S7). The introduction of water molecules in MD simulations may have created significant hydrogen bond interactions towards the ligand, pulling it from the binding pocket. The induced dipole interaction of the hydroxyl or methoxy groups against the binding pocket may have been insufficient to keep the ligands in the cavity.

C-0310, C-0829, C-1757, and C-1933 formed stable complexes with minor conformational changes, evidenced by their low and steady ligand–RMSD values (Figure 10). C-1254 and C-2082 showed small fluctuations in ligand–RMSD, suggesting that their initial docking configuration shifted to a slightly different binding pose. Still, these ligands reached definite and stable binding poses, albeit ones that were somewhat different from their starting conformations. The highly rotatable structure of CS9 and the flexible phenyl and alkyl groups of C-1704 caused fluctuations in their ligand–RMSD. Meanwhile, the three unstable flavanones that moved out of the protein’s binding pocket showed minor ligand–RMSD changes because of their more compact structures.

No significant effect on RMSF plots was observed due to the dissociation of the unstable ligand (Figure 11). The active site residues’ RMSF values remained similar to those obtained by stable complex residues, and the position of active site residues remained unchanged, regardless of whether the ligand was in the binding pocket or not. The high RMSF value from the residues 50 to 70 is from the flexible and nondescript loop that connects two alpha helices (Figure S8). It is freely exposed to the solvent and has no interactions with the other parts of the protein. 

C-0829, C-1704, C-1757, and C-1933 consistently had around three to four H-bond pairs within the binding site, similar to the three references (Figure 12). C-1254 produced around three to four H-bond pairs in the first 30 nanoseconds, but the number decreased as the simulation continued. C-2082 had the fewest H-bond interactions but did not affect the ligand’s overall stability inside the binding cavity. The ligands that moved out of the binding site did not form any H-bond interactions with the protein’s active site. The H-bond interactions observed among the unstable ligands towards the middle of the simulation were only temporary, caused by the ligands randomly attaching to the protein’s surface.

### 2.5. MM/PBSA Analysis

**Figure 13:**
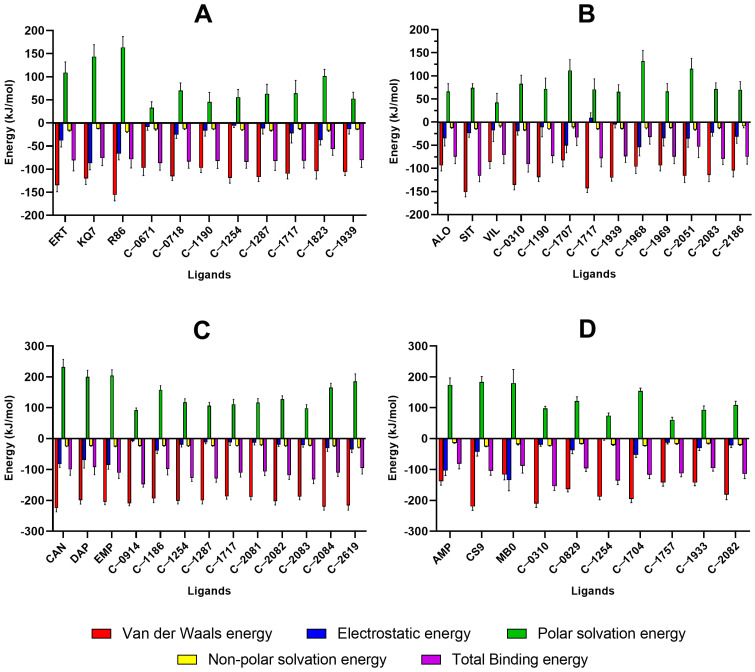
Average binding energies of the potential natural inhibitors against the four protein targets using MM/PBSA: (**A**) PTP1B, (**B**) DPP-4, (**C**) SGLT-2, (**D**) FBPase. C-0888 and C-1847 from the PTP1B complexes; and C-0690, C-1433, and C-1883 from FBPase complexes are excluded because of their unstable behavior with their respective proteins. The binding energy is estimated from the sum of the van der Waals, electrostatic, polar solvation, and non-polar solvation energies during the last 10 ns of the simulation.

**Figure 14:**
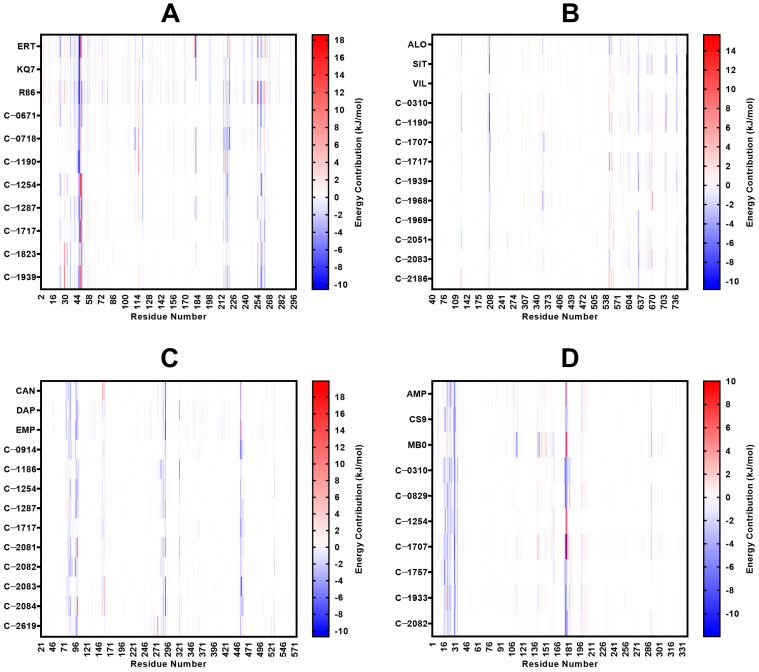
The energy contribution of the protein residues in the binding site between the ligand and the receptor: (**A**) PTP1B, (**B**) DPP-4, (**C**) SGLT-2, (**D**) FBPase. Attractive and repulsive interactions are represented by blue and red streaks, respectively. There is an apparent similarity between the energies imparted by the protein residues when interacting with the three references and the candidate ligands. Attractive forces are concentrated towards residues Arg 45, Tyr 46, Tyr 47, Ala 217, Lys 120, Ile 219, Met 258, and Gln 262 for PTP1B; towards Glu 205, Glu 206, Tyr 547, and Trp 629 for DPP-4; towards Phe 98, Phe 453, Val 95, Val 157, Leu 283, Tyr 290, and Leu 84 for SGLT-2; and towards Val 17, Met 18, Gly 21, Ala 24, Leu 30, and Met 177 for FBPase.

The complexes that behaved stably were subjected to MM/PBSA analysis to evaluate the binding energy between the ligand and the protein. Results are shown in Figure 13 and Tables S4–S7 in the Supplementary Materials. The binding free energy of C-1254 and C-1717 were observed to be equally potent compared to the reference drugs (Figure 13), indicating that both compounds have the potential to be as effective as the currently established drugs. Moreover, due to their multi-target nature, these compounds may exhibit more effective glucose-lowering effects than the currently available options. Both compounds also presented attractive interactions with the known crucial residues of their respective proteins (Figure 14). Specifically, C-1254 demonstrated attractive interactions with the critical residues of PTP1B, SGLT-2, and FBPase, while C-1717 displayed favorable interactions with PTP1B, DPP-4, and SGLT-2. The compounds targeting two proteins (C-0310, C-1190, C-1287, C-1939, C-2082, and C-2083) also scored favorable binding energies. 

#### 2.5.1. PTP1B

For the PTP1B complexes, C-0671 obtained the strongest binding energy (−87.20 kJ/mol), followed by C-1254 (−84.32 kJ/mol) and C-0718 (−83.73 kJ/mol). C-1823 is the only candidate ligand with a weaker binding score (−56.60 kJ/mol) than any three control compounds. Although it did not surpass the scores of the references, C-1823 still favors complex formation because of its negative binding energy, but its binding strength is not as strong as the three reference compounds of PTP1B. Van der Waals energy contributed the most to the binding strength of all ligands, while electrostatic interactions were more significant towards KQ7, R86, and C-1823 due to their extensive network of H-bonds. Because of C-1823’s low MM/PBSA score, it was omitted from the list of potential candidate ligands. 

The residues Arg 45, Tyr 46, Tyr 47, Ala 217, Lys 120, Ile 219, Met 258, and Gln 262 imparted the most substantial binding strength in the protein’s binding pocket (Figure 14). These residues encompass all four binding subsites in the protein’s catalytic domain, which could enhance the inhibitor’s potency and selectivity [33,42]. Lys 120 is a crucial PTP1B residue that significantly enhances ligand potency and selectivity, while Tyr 47 and Gln 262 are integral parts of the binding pocket for some compounds [30,41], so the residues’ strong, attractive interactions may also improve the inhibitor’s potency. Tyr 46, Arg 45, Ile 219, and Met 258 recorded the highest energy contribution in almost all top candidate ligands (Figure S9). Tyr 46 and Met 258 constantly had the strongest molecular mechanics energy contributions, compensating for the positive polar solvation energies. Arg 24 and Arg 45 had favorable polar solvation energies, and in some protein–ligand systems, the solvation energies were stronger than the combined electrostatic and van der Waals energies.

#### 2.5.2. DPP-4

Three stable ligands (C-1707, C-1968, and C-2051) bound to DPP-4 had weaker binding energies (−34.93 kJ/mol to −59.44 kJ/mol) than the three reference compounds (−71.05 kJ/mol to −116.44 kJ/mol). C-2083 recorded the highest MM/PBSA score (−114.47 kJ/mol), but none of the 10 candidate ligands obtained a better score than the reference SIT (−116.44 kJ/mol). The strong van der Waals energies prevented the complex’s tendency to dissociate as a result of the positive polar solvation energies. The highest van der Waals energies were observed with C-0310, C-2083, and C-1717. C-2083 initially had three different binding interactions with the receptor, and its consistent H-bond networks contributed significantly to its binding strength. C-1707 had strong electrostatic interactions due to its polar structure, but its weak van der Waals forces and positive polar solvation energy led to weak binding. Similarly, C-1968 and C-2051 had low MM/PBSA scores due to their positive polar solvation energies, and the dwindling number of H-bonds in the C-2051 complex may have affected their binding strength. The presence of hydroxyl groups around their structures led to higher polar surface areas, resulting in stronger electrostatic interactions but also more positive polar solvation energies. 

The residues Glu 205, Glu 206, Tyr 547, and Trp 629 imparted the most significant energy contribution to the binding activity (Figure 13). Asp 708 and His 740 also had favorable connections in some ligands, though they were not as strong as the first five residues. Studies have shown that Glu 205 and Glu 206 strongly interact with known inhibitors of DPP-4 and are crucial for enzymatic activity [34,36]. Similarly, Tyr 547 and Trp 629 play important roles in stabilizing the binding and forming extensive binding networks [44]. As such, the ligands’ interaction with these residues may significantly improve the potency and the binding activity. Asp 708 and His 740 are part of the catalytic triad, so binding to these residues will likely impede the protein’s catalytic activity. Tyr 547 and Trp 629 had the most negative energy contributions in most potential ligands (Figure S10). Despite their tendency to favor complex dissociation due to their positive polar solvation energies, Tyr 547 and Trp 629 consistently produced strong molecular mechanics interactions that compensated for the unfavorable binding energy caused by solvation.

#### 2.5.3. SGLT-2

The calculated binding energies of all ten candidate ligands were found to be more potent than at least one of the reference compounds (Figure 13). Seven natural compounds obtained a more negative binding score (−110.71 kJ/mol to −147.29 kJ/mol) than any of the three references (−91.70 kJ/mol to −110.64 kJ/mol). C-0914 registered the highest binding score, albeit with weak electrostatic interaction from the low number of H-bond connections. C-1186, C-2084, and C-2619 had the most polar moieties and generated consistent H-bond pairs resulting in relatively strong electrostatic interactions. 

Interaction with Phe 98 and Phe 453 of the human SGLT-2 proteins has been reported to drive Empagliflozin’s potency, while Val 95, Val 157, and Leu 283 improve its selectivity [39]. From Figure 14, Phe 453, Phe 98, and Leu 283 exhibited some of the strongest attractive interactions in all ten candidate ligands and the three references. At the same time, Val 157 and Val 95 imparted modest energy contributions, further highlighting their importance in the binding activity. Other residues with notable contributions to binding include Tyr 290, Leu 84, Ile 456, Gln 457, Leu 283, and Leu 527. Tyr 290, Phe 453, Phe 98, and Leu 84 are among the top binding energy contributors in most ligands. His 80, Tyr 290, and Gln 457 are not included in the critical residues targeted by Empagliflozin but present integral roles in binding, providing strong van der Waals and electrostatic interactions that offset the large positive polar solvation energies. Meanwhile, non-polar solvation energies are insufficient to compensate for the rise in repulsive forces during solvation.

#### 2.5.4. FBPase

The remaining seven FBPase candidate inhibitors displayed better affinity towards the protein than at least one reference compound (Figure 13). Only the MM/PBSA scores of C-0829 (−95.90 kJ/mol) and C-1933 (−95.49 kJ/mol) did not surpass the score of the reference CS9 (−103.60 kJ/mol), whereas the rest exhibited stronger binding affinity than all three control compounds (−80.82 kJ/mol to −103.60 kJ/mol). C-1254 and C-0310 obtained the most negative binding energies, despite receiving the weakest electrostatic interactions, due to having only one hydroxyl group, unlike the highly polar reference compounds. C-1933 had strong electrostatic interactions but weak van der Waals energy. Nevertheless, its MM/PBSA score was still higher than that of the reference AMP. Similar to the three previous protein systems, the total solvation energy favored complex dissociation because of the positive polar solvation energies and weak non-polar energy contributions. 

Studies have pointed out the importance of Lys 112, Tyr 113, Met 177, and Thr 31 because of the observed decrease in inhibitory potency when these residues are mutated [40,41]. Met 177 displayed the most prominent contribution to binding, while Lys 112, Tyr 113, and Thr 31 had modest contributions (Figure 14). Other residues with significant binding strength against the candidate ligands and the references include Val 17, Leu 30, Ala 24, and Gly 21—all of which are situated inside the protein’s binding pocket [17,45]. The combined polar and non-polar solvation energies favored protein–ligand separation but were counteracted by strong intermolecular forces. As in previous proteins, molecular mechanics energy was the primary driving force for complex formation. Met 177, Leu 30, and Ala 24 were observed to have participated the most in all candidate ligands, emphasizing their significance in inhibiting the protein (Figure S12).

### 2.6. Identification of Top Inhibitor

Table 3 and Table 4 present an improved ranking of the remaining candidate ligands for each protein target through a consensus scoring. This ranking was determined by combining the scores of the average complex–RMSD and MM/PBSA binding energy. The average complex–RMSD serves as an indicator of the complex’s stability. A lower average complex–RMSD indicates minimal structural changes from the initial docking pose and suggests minimal trajectory fluctuations, while a lower or more negative MM/PBSA binding energy signifies stronger binding affinity. Despite having different units, both parameters require smaller values to indicate stability or stronger binding affinity; therefore, the addition of both parameters served as a reasonable measure to identify the top inhibitor and fulfill the purpose of ranking. The highest-ranked compound for each protein represents the most promising candidate with the greatest potential to inhibit its respective target.

Based on Table 3 and Table 4, the compounds with the highest inhibitory potential against the four proteins are C-0671 against PTP1B, C-2083 against DPP-4, C-0914 against SGLT-2, and C-1254 against FBPase. These compounds exhibit excellent stability and binding affinity, suggesting their ability to effectively block the activity of their respective proteins. They also presented favorable interactions with the known critical residues of the protein targets. These findings highlight the potential of these four compounds as promising lead compounds or structural templates for the development of therapeutic agents against type 2 diabetes.

### 2.7. Plant Identification

Table 5 summarizes the list of phytochemicals that could inhibit the activity of the four therapeutic targets of diabetes. These compounds possess justified antagonistic characteristics, reflected by their stable RMSD trajectories and strong binding energies. Along with their excellent ADMET properties, these natural compounds have the potential to be developed into a safe and potent antidiabetic drug. 

Plants that contain at least one natural inhibitor from each protein group were also identified (Table 6) using the curated database of Indian Medicinal Plants, Phytochemistry, And Therapeutics (IMPPAT) [46] and PubChem [29]. Since the plants carry phytochemicals that can inhibit each of the four targets, the purified plant extract containing the identified hit compounds may work synergistically by simultaneously targeting multiple pathways, thereby enhancing the ability to lower glucose levels.

Most of the plants listed in Table 6 exhibited antidiabetic potential from some in vivo experiments. Leaf suspension of *Eclipta prostata* was administered to streptozotocin-induced diabetic rats and lowered blood glucose levels by 17.6% [64]. It also resulted in a decreased activity in glucose-6-phosphatase and fructose 1,6-bisphosphatase, both precursors for forming free glucose in the gluconeogenesis process catalyzed by FBPase [65]. In another study, the proponents confirmed the hypoglycemic effects of *Agave sisalana*, but could not identify the specific key ingredient from the observed antidiabetic activity [66]. No studies have investigated the antihyperglycemic potential of *Piper anducum,* but the antidiabetic potential of *Piper claussenianum*, a closely related plant from the same family, was studied. Methanolic extracts of *Piper claussenianum* flowers given to streptozotocin-induced diabetic rats reduced glucose levels from 318.4 ± 28.1 mg/dl to 174.2 ± 38.3 mg/dl after 12 days of treatment. The researchers isolated three compounds from the extract (i.e., chalcone, pinocembrin, and alpinetin) and identified chalcone as the main key player in lowering blood glucose. However, they failed to specify the specific biological mechanisms of how chalcone contributes to hypoglycemic activity [67]. In a study by Madhavan et al., *Curculigo orchioides* root tubers were used to treat diabetes in alloxan-induced diabetic rats. The plant extract successfully attenuated blood glucose levels from 300.47 mg/dl to 142.40 mg/dl, or a 53.92% reduction, after 21 days of administration [68]. Similar glucose-lowering results were also observed when *Luffa cylindrica* fruit extract was given to alloxan-induced diabetic rats, but neither the key phytochemical nor the mechanism of hypoglycemic activity were not discussed [69,70]. For *Moringa oleifera*, *Alium cepa*, and *Helianthus annuus*, our in silico simulations revealed sterols (i.e., brassicasterol, campesterol, stigmasterol, and sitosterol) as the main key players in inhibiting the four protein targets. Many in vivo studies have validated the hypoglycemic effects of phytosterols [71,72,73], which supports the observed inhibition of the four protein targets using in silico methods. Some sterols have been shown to prevent the breakdown of starch to simple sugar by inhibiting the α-glucosidase enzyme, but other signaling pathways related to diabetes management have not been explored [73]. Further clinical studies are required to confirm the potential therapeutic effects of plant sterols in mitigating symptoms associated with diabetes.

## 3. Materials and Methods

### 3.1. ADMET Profiling

A total of 2657 phytochemicals from Filipino plants were downloaded from the curated database of the Indian Medicinal Plants, Phytochemistry And Therapeutics (IMPPAT) (https://cb.imsc.res.in/imppat/ (accessed on 24 January 2022)) [46], Dr. Duke’s Phytochemical and Ethnobotanical Databases (https://phytochem.nal.usda.gov (accessed on 24 January 2022)) [51], and PubChem (https://pubchem.ncbi.nlm.nih.gov/ (accessed on 24 January 2022)) [29]. Three reference compounds for each protein target were also included: ertiprotafib (ERT), KQ-791 (KQ7), and 864135-09-1 CID: 11786814 (R86) for PTP1B [33,74]; alogliptin (ALO), sitagliptin (SIT), and vildagliptin (VIL) for DPP-4 [13]; canagliflozin (CAN), dapagliflozin (DAP), and empagliflozin (EMP) for SGLT-2 [14]; and adenosine monophosphate (AMP), CS-917 (CS9), and MB07803 (MB0) for FBPase [27]. The absorption, distribution, metabolism, excretion, and toxicity (ADMET) profile of all 2657 natural compounds and 12 reference compounds were analyzed using the ADMETLab 2.0 server (https://admetmesh.scbdd.com (accessed on 7 February 2022)) to predict the natural compounds’ pharmacokinetic and toxicity properties [75]. Six of the most important and commonly used parameters in drug development studies of oral medicines were assessed: (1) Lipinski’s rule of five, a general rule of thumb to evaluate drug-likeness, (2) Human intestinal absorption, a fundamental parameter that influences bioavailability [76], (3) Human oral bioavailability, one of the essential pharmacokinetic properties and an indicator of how effectively the medicine is delivered to the systemic circulation, [75] (4) Carcinogenicity, (5) Hepatoxicity, and (6) Acute toxicity rule, which identifies molecules that contain toxic moieties or functional groups.

### 3.2. Ligand Structure Optimization Using DFT

Quantum Espresso [77] and BURAI (https://burai.readthedocs.io/en/latest/ (accessed on 10 February 2022)) were used for the fixed-cell geometry optimization of the ligands in an orthorhombic lattice. The software utilized a plane-wave basis set and pseudopotentials, with the Broyden, Fletcher, Goldfarb, and Shanno (BFGS) algorithm for the structural optimization. To ensure convergence, the number of steps and time duration were set to 200 and 2 days, respectively. The kinetic energy cutoff for wavefunctions was 25 Ry, while the kinetic energy cutoff for the charge density and potential was 225 Ry. All convergence thresholds for total energy, forces, ion optimization, and self-consistency were kept at their default values. 

### 3.3. Protein Optimization

The structures of the proteins were downloaded from the RCSB PDB database (rcsb.org) [78]: PTP1B (PDB ID: 2QBP), DPP-4 (PDB ID: 2ONC), SGLT-2 (PDB ID: 7VSI), FBPase (PDB ID: 2FIE). Water ions, co-crystallized ligands, and other unneeded molecules were removed from the proteins using Maestro [79]. The clean PDB files were then subjected to MODELLER, where a series of Python codes supplied missing residues and refined the loops generated [80]. Since new loops were added, a minimization step was performed using the steepest descent algorithm to ensure that the protein structure was relaxed before the docking experiments.

### 3.4. Molecular Docking

The optimized structures of the ligands were subjected to two molecular docking experiments using Autodock 4.2.6 [31] and Autodock Vina 1.2.3 [32]. The known residues surrounding the proteins’ binding pocket were used as a guide to locate their respective active sites [34,39,45,81]. Using AutodockTools 1.5.7 [31], polar hydrogens were first added to the protein, and Kollman charges were also assigned to represent their partial positive or negative charge distributions. These charges were derived from the corresponding electrostatic potential using ab initio quantum mechanical calculations [82]. The structure of the ligands and the protein were saved as PDBQT files containing the coordinates of the atoms, the partial charges, and the atom types. Both docking software packages utilized a 60 × 60 × 60 grid box to enclose the binding site and to keep the ligands within the centroid of the known residues. Protein residues were kept rigid, and only the rotatable bonds of the ligands were allowed to move. In Autodock 4.2.6, the Lamarckian genetic algorithm with 1000 runs was used to predict the binding poses of the ligand. The Python codes prepare_gp4.py, and prepare_dp4.py were used to generate the grid parameter files (GPF) and docking parameter files (GPF). For Autodock Vina 1.2.3, the number of runs (exhaustiveness) and the number of poses (num_modes) were both set to 1000. Similar to Autodock 4.2.6, Vina also uses empirical scoring functions, but its scoring methodology follows more of a machine-learning approach than the force field-based scoring functions of Autodock 4.2.6 [83]. Consensus docking was then implemented to refine the molecular docking results. Structure-based virtual screening heavily relies on the scoring function, so it was important to apply methods that could improve the reliability and accuracy of the results [30]. The RMSD between the poses with the most negative binding score from Autodock 4.2.6 and Vina was first estimated. If the RMSD value was less than 2 Å, the ligand proceeded to the next step; otherwise, the ligand was rejected. AutodockTools 1.5.7 was used to calculate the RMSDs of the docked poses. 

### 3.5. Molecular Dynamics (MD) Simulation

Molecular dynamics (MD) simulations were performed on the top 10 ligands with the highest docking scores using GROMACS 2021-4 [84] to determine the stability of protein–ligand complexes. The topology of the protein was built using the July 2021 version of Chemistry at Harvard Macromolecular Mechanics (CHARMM) 36 force field [85], while the ligands’ force field parameters were generated from the CGenFF (https://cgenff.umaryland.edu/ (accessed on 28 February 2022)) server of the University of Maryland—Baltimore [86]. The protonation states of the residues were assigned using the GROMACS command pdb2gmx, assuming canonical pKa values and a pH of 7. The system was enclosed inside a cubic box with periodic boundary conditions, 10 Å from the protein’s edge. The box was solvated with TIP3P water molecules, and sodium and chlorine ions were added to neutralize the system. After neutralization, more sodium and chlorine ions were added to mimic the human physiological condition of 0.15 M. This was followed by a 5000-step energy minimization using the steepest descent algorithm to relax the system and ensure that there were no steric clashes. Next, equilibration was conducted via two 100-ps simulation runs to optimize the solvent with the protein–ligand complex. The first phase was carried out under a canonical NVT ensemble where the number of particles, volume, and temperature were kept constant. This step aimed to bring the system’s temperature to the desired temperature of 310 K. After the temperature had stabilized, equilibration of pressure under an NPT ensemble or under a constant number of particles, pressure, and temperature was conducted to reach the desired pressure of 1 bar. The temperature and pressure of the system was controlled and regulated using the modified Berendsen thermostat and Berendsen barostat, respectively. The neighbor list was updated every 20 steps using the Verlet neighbor search algorithm. Van der Waals and electrostatic cutoff distances were kept at 1.2 nm, and the Particle Mesh Ewald method was employed to compute the long-range electrostatic interactions. Once the system had equilibrated at the desired temperature and pressure, a 100 ns MD production run was performed under NPT ensemble. The MD simulation employed a timestep of 2 fs, resulting in a total of 50,000,000 evaluations. During the run, 1000 frames were saved at intervals of 100 ps.

### 3.6. Molecular Mechanics/Poisson–Boltzmann Surface Area (MM/PBSA) Calculation

The molecular mechanics/Poisson–Boltzmann surface area (MM/PBSA) approach was used to predict the protein–ligand binding affinity by combining molecular mechanics calculations and continuum solvation models. The MM/PBSA scores were estimated using the GROMACS-compatible g_mmpbsa package developed by Kumari et al. The Python scripts MmPbSaStat.py and MmPbSaDecomp.py calculated the energies during the last 10 ns of the simulation and decomposed the binding free energy on a per-residue basis, respectively [87]. 

The method utilizes Coulomb’s Law and Lennard–Jones potential functions to calculate the electrostatic and van der Waals interaction energies, respectively [81]. Polar solvation energies are estimated by solving the Poisson-Boltzmann (PB) equation, while, for the non-polar contribution of the solvation energy, it is assumed that the energy due to cavity formation is linearly dependent on the surface accessible surface area (SASA). The g_mmpbsa tool does not compute entropic energy, so only relative binding energies are provided, which are suitable for comparing the binding strengths of ligands to the same receptor. Entropic energy calculations are computationally expensive, time-consuming, and often contain large errors making the results less reliable [87,88]. Since these do not improve the results, the entropy term has been omitted in several MM/PBSA studies [89]. The binding strength between the ligand and protein can therefore be estimated using the following simplified formula:

(1) $$∆G_{bind}={∆E_{vdW}+∆E}_{elec}+{∆G}_{PB}+{∆G}_{SASA}$$

## 4. Conclusions

Natural compounds from Filipino plants were screened for their inhibitory potential against four diabetes therapeutic targets using a wide array of computer-aided drug discovery techniques. Out of the 2657 compounds screened, 373 passed the ADMET screening and proceeded to molecular docking to predict and rank the binding poses. The top ten highest-scoring ligands from each protein target were then subjected to 100 ns MD simulations to assess the stability and binding strengths of the complexes. From the ten candidate ligands from each protein target, we identified seven natural compounds against PTP1B, seven against DPP-4, ten against SGLT-2, and seven against FBPase, each possessing justified antagonistic characteristics to inhibit their respective protein receptors. Among the top candidates, the compounds gypsogenin (C-0671) against PTP1B, adunctin C (C-2083) against DPP-4, sitosterol (C-0914) against SGLT-2, and stigmasterol (C-1254) against FBPase exhibited the most promising inhibitory characteristics. The RMSD and RMSF trajectories elucidated the stable behaviors of these compounds, while the MM/PBSA analysis revealed the top ligands with binding affinity comparable to the reference compounds. Detailed interaction analysis and energy contribution breakdown also revealed the key residues integral to the binding activity: Arg 45, Tyr 46, Tyr 47, Ala 217, Lys 120, Ile 219, Met 258, and Gln 262 for PTP1B; Glu 205, Glu 206, Tyr 547, and Trp 629 for DPP-4; Phe 98, Phe 453, Val 95, Val 157, Leu 283, Tyr 290, and Leu 84 for SGLT,2; and Val 17, Met 18, Gly 21, Ala 24, Leu 30, and Met 177 for FBPase. We recommend that these phytochemicals be further investigated in future in vivo and in vitro experiments to verify their viability as inhibitor drugs. Finally, we identified eight plants in the Philippines (*Eclipta prostata*, *Agave sisalana*, *Piper aduncum*, *Curculigo orchioides*, *Luffa cylindrica*, *Moringa oleifera*, *Alium cepa*, and *Helianthus annuus*) carrying at least one natural inhibitor against each protein target. The phytochemical stigmasterol (C-1254) was found in all plants that effectively target three proteins: PTP1B, SGLT-2, and FBPase. Additionally, the plant *Eclipta prostata* contains the compound 4beta-hydroxyverazine (C-1717), which has been observed to inhibit three proteins: PTP1B, DPP-4, and SGLT-2. Furthermore, several other phytochemicals, including brassicasterol, campesterol, yuccagenin, 9-dehydrohecogenin, adunctin B, and adunctin C, have demonstrated the ability to target a maximum of two proteins. The eight plants are also recommended to be further investigated in the next stage of the drug discovery pipeline to determine the synergetic effects of the natural inhibitors found in the plant against the four targets. The purified extract containing the key phytochemicals could be developed into a safe, cost-effective, and scientifically validated multi-target drug that may improve the efficacy of existing diabetes treatments. 

## Figures and Tables

**Table 1:** The number of ligands that passed or failed in each ADMET property.

ADMET Property	Passed	Failed
Human Intestinal Absorption	1994	663
Human Oral Bioavailability	984	1673
Carcinogenicity	1962	695
Hepatoxicity	1641	1016
Acute Toxicity Rule	2648	9
Lipinski’s Rule of Five	2187	470
All six parameters	373	2284

**Table 2:** Ranking of the top 10 ligands with the highest docking scores against PTP1B, DPP-4, SGLT-2, and FBPase. The consolidated docking scores are the sum of the scores from Autodock 4.2 and Autodock Vina. References used are italicized: Ertiprotafib (ERT), KQ-791(KQ7), 864135-09-1 CID: 11786814 (R86), Alogliptin (ALO), Sitagliptin (SIT), Vildagliptin (VIL), Canagliflozin (CAN), Dapagliflozin (DAP), Empagliflozin (EMP), MB06322 or CS-917 (CS9), MB07803 (MB0), and adenosine 5′-monophosphate (AMP).

	PTP1B		DPP-4		SGLT-2		FBPase	
Rank	Compound	Consensus Docking Score (kJ/mol)	Compound	Consensus Docking Score (kJ/mol)	Compound	Consensus Docking Score (kJ/mol)	Compound	Consensus Docking Score (kJ/mol)
	*ERT* *	*−79.08*	*ALO*	*−80.79*	*CAN*	*−96.06*	*CS9* *	*−60.46*
	*KQ7* *	*−75.77*	*SIT*	*−75.44*	*DAP*	*−87.11*	*MB0* *	*−58.87*
	*R86* *	*−96.61*	*VIL*	*−70.33*	*EMP*	*−92.17*	*AMP* *	*−59.96*
1	C-1823	−81.96	C-1939	−83.85	C-1254	−94.43	C-0829	−67.53
2	C-0671	−81.50	C-1968	−82.30	C-2084	−94.22	C-1757	−66.23
3	C-0718	−79.08	C-2186	−81.88	C-2083	−91.46	C-1433	−64.35
4	C-1254	−77.49	C-1190	−80.42	C-1186	−91.21	C-1254	−64.27
5	C-1847	−77.40	C-0310	−78.91	C-1287	−89.24	C-2082	−64.14
6	C-0888	−77.24	C-1717	−78.70	C-0914	−89.16	C-0310	−63.93
7	C-1287	−76.02	C-1707	−78.49	C-2082	−88.37	C-1704	−63.51
8	C-1190	−75.56	C-2083	−76.27	C-2619	−87.82	C-0690	−63.47
9	C-1939	−74.77	C-2051	−76.23	C-2081	−87.70	C-1933	−63.47
10	C-1717	−74.39	C-1969	−75.94	C-1717	−87.40	C-1883	−62.97

Italicized texts are the scores of the reference compounds; * not commercially available.

**Table 3:** Consensus ranking of the top candidate ligands against PTP1B and DPP-4 using the average complex–RMSD and MM/PBSA binding energies.

PTP1B							DPP-4						
Compound	Average Complex–RMSD	Stability Rank	Binding Energy (kJ/mol)	Binding Affinity Rank	MD Consensus Score	Overall Rank	Compound	Average Complex–RMSD	Stability Rank	Binding Energy (kJ/mol)	Binding Affinity Rank	MD Consensus Score	Overall Rank
C-0671	2.230	1	−19.279	1	−17.049	1	C-2083	2.508	4	−22.289	1	−19.781	1
C-1287	2.319	5	−19.163	2	−16.843	2	C-1969	2.395	2	−19.235	2	−16.841	2
C-0718	2.304	3	−18.641	4	−16.337	3	C-0310	2.132	1	−18.867	3	−16.735	3
C-1939	2.371	6	−18.666	3	−16.295	4	C-2186	2.711	6	−17.731	4	−15.020	4
C-1254	2.275	2	−18.533	5	−16.258	5	C-1717	2.464	3	−17.185	7	−14.722	5
C-1717	2.315	4	−18.229	6	−15.915	6	C-1939	2.999	7	−17.551	5	−14.552	6
C-1190	2.638	7	−17.872	7	−15.234	7	C-1190	2.685	5	−17.204	6	−14.519	7

**Table 4:** Consensus ranking of the top candidate ligands against SGLT-2 and FBPase using the average complex–RMSD and MM/PBSA binding energies.

SGLT-2							FBPase						
Compound	Average Complex–RMSD	Stability Rank	Binding Energy (kJ/mol)	Binding Affinity Rank	MD Consensus Score	Overall Rank	Compound	Average Complex–RMSD	Stability Rank	Binding Energy (kJ/mol)	Binding Affinity Rank	MD Consensus Score	Overall Rank
C-0914	3.113	8	−34.815	1	−31.702	1	C-1254	2.415	6	−31.798	1	−29.384	1
C-2083	2.977	2	−30.268	2	−27.291	2	C-0310	2.499	7	−28.676	2	−26.177	2
C-1287	3.008	4	−29.796	3	−26.788	3	C-1757	2.262	2	−25.427	3	−23.165	3
C-2082	3.215	10	−27.630	4	−24.415	4	C-2082	2.401	4	−25.289	4	−22.888	4
C-1254	3.064	6	−27.183	5	−24.119	5	C-1704	2.410	5	−22.591	5	−20.181	5
C-2081	3.050	5	−25.333	6	−22.282	6	C-0829	2.236	1	−22.380	6	−20.144	6
C-1717	3.070	7	−24.787	7	−21.717	7	C-1933	2.339	3	−17.946	7	−15.607	7
C-1186	2.981	3	−24.065	8	−21.084	8							
C-2084	3.118	9	−23.333	9	−20.215	9							
C-2619	2.920	1	−22.378	10	−19.458	10							

**Table 5:** Final list of phytochemicals with antagonistic potential to inhibit the four therapeutic targets of diabetes.

PTP1B		DPP-4		SGLT-2		FBPase	
C-0671	Gypsogenin	C-0310	Campesterol	C-0914	Sitosterol	C-0310	Campesterol
C-0718	Quillaic acid	C-1190	Yuccagenin	C-1186	Saroaspidin B	C-0829	Actinodaphnine
C-1190	Yuccagenin	C-1717	4beta-Hydroxyverazine	C-1254	Stigmasterol	C-1254	Stigmasterol
C-1254	Stigmasterol	C-1939	9-Dehydrohecogenin	C-1287	Brassicasterol	C-1704	Piperaduncin A
C-1287	Brassicasterol	C-1969	Veramiline	C-1717	4beta-Hydroxyverazine	C-1757	Copalic Acid
C-1717	4beta-Hydroxyverazine	C-2083	Adunctin C	C-2081	Adunctin A	C-1933	Norisoboldine
C-1939	9-Dehydrohecogenin	C-2186	Hongguanggenin	C-2082	Adunctin B	C-2082	Adunctin B
				C-2083	Adunctin C		
				C-2084	Adunctin E		
				C-2619	Deserpidine		

**Table 6:** List of plants with potential inhibitory activity against four therapeutic targets of diabetes.

Plant	Part	Protein Target	Phytochemical	Reference
*Eclipta prostata*	Leaves	PTP1B	4beta-Hydroxyverazine	[47]
			Stigmasterol	[48]
		DPP-4	4beta-Hydroxyverazine	[47]
			Veramiline	[47]
		SGLT-2	4beta-Hydroxyverazine	[47]
			Stigmasterol	[48]
			Sitosterol	[48]
		FBPase	Stigmasterol	[48]
*Agave sisalana*	Leaves	PTP1B	9-Dehydrohecogenin	[49]
			Stigmasterol	[50]
		DPP-4	9-Dehydrohecogenin	[49]
			Hongguanggenin	[51]
		SGLT-2	Stigmasterol	[50]
			Sitosterol	[50]
		FBPase	Stigmasterol	[50]
			Campesterol	[50]
*Piper aduncum*	Leaves	PTP1B	Stigmasterol	[52]
		DPP-4	Adunctin C	[53]
		SGLT-2	Adunctin A	[53]
			Adunctin B	[53]
			Adunctin C	[53]
			Adunctin E	[53]
			Stigmasterol	[52]
		FBPase	Adunctin B	[53]
			Stigmasterol	[52]
			Piperaduncin A	[54]
*Curculigo orchioides*	Rhizomes	PTP1B	Yuccagenin	[55]
			Stigmasterol	[55]
		DPP-4	Yuccagenin	[55]
		SGLT-2	Stigmasterol	[55]
			Sitosterol	[55]
		FBPase	Stigmasterol	[55]
*Luffa cylindrica*	Seeds	PTP1B	Gypsogenin	[56]
			Quillaic Acid	[57]
			Stigmasterol	[58]
		DPP-4	Campesterol	[59]
*Luffa cylindrica*	Seeds	SGLT-2	Stigmasterol	[58]
			Sitosterol	[58]
		FBPase	Stigmasterol	[58]
			Campesterol	[59]
*Moringa oleifera*	Seeds	PTP1B	Brassicasterol	[60,61]
			Stigmasterol	[60,61]
		DPP-4	Campesterol	[60,61]
		SGLT-2	Brassicasterol	[60,61]
			Stigmasterol	[60,61]
		FBPase	Stigmasterol	[60,61]
			Campesterol	[60,61]
*Alium cepa*	Bulb	PTP1B	Brassicasterol	[62]
			Stigmasterol	[62]
		DPP-4	Campesterol	[62]
		SGLT-2	Brassicasterol	[62]
			Stigmasterol	[62]
			Sitosterol	[62]
		FBPase	Stigmasterol	[62]
			Campesterol	[62]
			Stigmasterol	[62]
*Helianthus annuus*	Seeds	PTP1B	Stigmasterol	[63]
		DPP-4	Campesterol	[63]
		SGLT-2	Stigmasterol	[63]
			Sitosterol	[63]
		FBPase	Stigmasterol	[63]
			Campesterol	[63]

## Data Availability

The data presented in this study are available in the article and the Supplementary Materials.

## Supplementary Materials

The following supporting information can be downloaded at https://doi.org/10.5281/zenodo.7949530. Table S1. List of ligands with favorable ADMET profile. Results are obtained from ADMETlab 2.0. Table S2. ADMET results of the control compounds used in this study. Table S3. Docking scores of the 373 ligands that passed the ADMET filter. Table S4. Average binding energies of the potential natural inhibitors against PTP1B using MM/PBSA. Table S5. Average binding energies of the potential natural inhibitors against DPP-4 using MM/PBSA. Table S6. Average binding energies of the potential natural inhibitors against SGLT-2 using MM/PBSA. Table S7. Average binding energies of the potential natural inhibitors against FBPase using MM/PBSA. Figure S1. Top ten highest-scoring ligands against PTP1B. Figure S2. Top ten highest-scoring ligands against DPP-4. Figure S3. Top ten highest-scoring ligands against SGLT-2. Figure S4. Top ten highest-scoring ligands against FBPase. Figure S5. The DPP-4 protein has a patternless loop formed by a group of amino acids from residues 240 to 260 exposed to the water solvent that caused the large peak observed in the RMSF plots. Figure S6. The noticeable high peaks in the RMSF plots of SGLT-2 complexes are from the partnerless loops connecting two alpha helices. Figure S7. Docking poses of C-0690, C-1433, and C-1883 against the binding pocket of FBPase. Hydroxyl and methoxy groups are situated away from the binding pocket and towards the solvent. Figure S8. The FBPase protein has a large patternless loop formed by a group of amino acids from residues 50 to 70 exposed to the water solvent that caused the large peak observed in the RMSF plots. Figure S9. Energetic breakdown of PTP1B residues with the strongest binding energy contribution.. Figure S10. Energetic breakdown of DPP-4 residues with the strongest binding energy contribution. Figure S11. Energetic breakdown of SGLT-2 residues with the strongest binding energy contribution. Figure S12. Energetic breakdown of FBPase residues with the strongest binding energy contribution. Video S1—Sample 100-ns simulation clip (C-0671) of a stable complex against PTP1B. Video S2—100-ns simulation clip of C-0888 which had an unstable behavior against PTP1B. Video S3—100-ns simulation clip of C-1847 which had an unstable behavior against PTP1B. Video S4—Sample 100-ns simulation clip (C-2083) of a stable complex against DPP-4. Video S5—Sample 100-ns simulation clip (C-0914) of a stable complex against SGLT-2. Video S6—Sample 100-ns simulation clip (C-1254) of a stable complex against FBPase. Video S7—100-ns simulation clip of C-0690 which had an unstable behavior against FBPase. Video S8—100-ns simulation clip of C-1433 which had an unstable behavior against FBPase. Video S9—100-ns simulation clip of C-1883 which had an unstable behavior against FBPase.
